# Influence of parents’ and caregivers’ characteristics on the outcomes of antiretroviral treatment in Ugandan children

**DOI:** 10.4102/phcfm.v3i1.267

**Published:** 2011-11-08

**Authors:** Ntambwe Malangu

**Affiliations:** 1 Department of Epidemiology, University of Limpopo (Medunsa Campus), South Africa

## Abstract

**Background:**

The purpose of this study was to investigate whether there was an association between characteristics of parents and caregivers, and the outcomes of antiretroviral treatment in children younger than 6 years treated at Mildmay Centre in Uganda.

**Methods:**

This study was a cross-sectional study based on the review of records. The records of children treated from January 2000 to July 2005 were included in the analysis as part of a larger study. Descriptive and inferential statistics were used in the analysis of data.

**Results:**

Of the 179 children, 57.3% were male, 53.4% were 4–5 years, and their median age was 4 years. The majority of children were cared for, in descending order, by their mothers, aunts, grandmothers, and fathers. Whilst 16.0% were orphans of both parents, 56.9% had one of their parents still alive. With regard to outcomes of antiretroviral treatment, it was found that a CD4 count of less than 15% was the most significant predictor of death, when treatment was initiated only at that late stage. When the influence of caregivers’ and parents’ characteristics on the outcomes of treatment were considered, the only factor that was associated significantly with clinical improvement was the ‘father's unknown human immunodeficiency virus (HIV) status’. The data show that when the father was alive, as well as when both parents were alive, the children had a better chance of survival.

**Conclusion:**

The nature of the relationship between caregivers and children on antiretroviral treatment, as well as the HIV and living status of their parents seem to have little positive influence on the clinical, immunological, and survival outcomes of the children on treatment. More studies are needed to investigate other characteristics and relationships that may influence the outcomes of treatment.

## Introduction

### Setting

Outcomes of antiretroviral treatment have been described in developed countries, where the settings of health care differ from the under-resourced settings of developing countries. It is important that the situation of developing countries is described so that appropriate interventions can be planned. A paper based on the same data has been published; and it provided details on the demographic characteristics and outcomes of the treatment.^1^ This paper is based on a re-analysis of the same data. This re-analysis was motivated by the fact that, to date, little is known about the influence of the caregivers’ and parents’ characteristics on the outcomes of antiretroviral treatment: in particular, on the clinical, immunological and survival outcomes of children on ART. Kipp and co-workers^2^ conducted interviews with family caregivers of AIDS patients in three rural districts in western Uganda and reported that most support or care for AIDS patients is provided by family, mainly the elderly women and young girls, friends and the churches. Ueyama has reported that, in Malawi, maternal orphans and double orphans tend to face higher mortality risks and lower schooling outcomes than paternal orphans and non-orphans do, with boys facing more severe negative impacts from orphanhood than girls.^3^ The above reports suggest that the care and nurturing provided to children with human immunodeficiency virus (HIV), and particularly to those who have been orphaned, may influence their health outcomes. Hence, the purpose of this study was to investigate whether there was any association between characteristics of parents and caregivers, and the outcomes of antiretroviral treatment in children younger than 6 years treated at Mildmay Centre in Uganda. The Mildmay Centre opened in 1999 in Kampala, the capital of Uganda. To date, it offers care to more than 21 000 men, women and children living with HIV; by the end of 2005, about 202 children had been treated.^1, 4^

### Contribution to field

This study investigated whether there was an association between the characteristics of parents and caregivers; and the outcomes of antiretroviral treatment in children. The findings may contribute to the understanding of factors to be taken into account for policy decisions about adoption and access to antiretroviral treatment for children.

## Ethical considerations

Approval to conduct this study was obtained from the Medunsa Campus Research and Ethics Committee of the University of Limpopo. Permission to access the patients’ records was obtained from Management at the Mildmay Centre.

## Methods

Data were collected by using a retrospective chart review. The records of children under 6 who were treated at the centre from January 2000 to July 2005 were included in the analysis. Inclusion criteria comprised: aged less than 6 years, initiation on treatment at Mildmay Centre, and on antiretroviral treatment for at least 12 months.

A number of 202 children were identified who had been treated during this period. Of these, 16 records could not be retrieved, whilst 7 records did not meet one of the inclusion criteria, in that these children had not completed at least 12 months on antiretroviral treatment. Hence, 179 records were included in the final sample.

A pre-tested data collection form was used to collect sociodemographic and clinical data. The data collected for the patients (or children) were their age and gender, their relationship to the caregiver, their orphanhood status, whether they had died (and the causes of death) or whether they had survived, their CD4 count and their bodyweights. Information was collected about the caregivers’ gender and HIV status; details on the education level and income of caregivers could not be located on the records.

Descriptive statistics were calculated and statistical testing was performed with SPSS software (version 17.0; SPSS, Chicago, IL, USA). A logistic regression was conducted to adjust for confounding. The odds ratios of dichotomised variables were calculated. A ratio of above 1 suggested a protective effect or association, whilst a ratio of below 1 suggested the opposite; where the odds ratio was equal to 1, it meant that there was no effect. Statistical significance was assessed based on a confidence interval around the odds ratio; an interval including 1 meant that there was no statistical significance. In particular, comparisons were made between caregivers, the HIV-status of parents, and the living status of parents.

## Results

Of the 179 children, 57.3% were male, 53.4% were 4–5 years old, and their median age was 4 years. Where data were available, the majority of children was cared for (in descending order) by their mothers, aunts, grandmothers, and fathers (Table 1). A few of them were cared for by their parents as well as by sisters, uncles, grandfathers and guardians.

**TABLE 1:** Characteristics of caregivers and parents of children in the study (*N* = 179).

Variables and outcome	*f*	%
**Caregiver (*n* = 179)**		
Mother	92	51.4
Aunt	30	16.8
Grandmother	27	15.1
Father	12	6.7
Others (Both parents, sister, grandfather, guardian)	18	10.1
**Living status parents (*n* = 144)**		
Both alive	39	27.1
Both dead	23	16.0
One alive	82	56.9
**Living status mother (*n* = 102)**		
Alive	92	90.2
Dead	10	9.8
**Living status father (*n* = 77)**		
Alive	20	26.0
Dead	57	74.0
**HIV status parents (*n* = 144)**		
Both HIV-positive	74	48.1
Discordant couple	22	14.3
Both unknown	58	37.7
**HIV status father (*n* = 77)**		
Unknown	23	29.9
HIV-positive	43	55.8
HIV-negative	11	14.3
**HIV status mother (*n* = 102)**		
Unknown	38	37.3
HIV-positive	60	58.8
HIV-negative	4	3.9

*Source*: Author's original data

*N*, total number of children; *n*, given as a means of number; *f*, frequency.

The sample comprised, inter alia, 16% of children whose parents both had died whilst 56.9% had one living parent. Amongst the latter group, numerous patients’ mothers were still living. The HIV status of some parents was known; about half of the parents were confirmed as HIV-positive. The HIV status was unknown, however, for 37.3% of mothers and 29.9% of fathers respectively.

With regard to clinical and immunological outcomes of antiretroviral treatment, there was a significant improvement in the patients’ bodyweights and CD4 count from baseline to the 6th month on treatment (Table 2). This improvement was influenced by factors such as the baseline immune status of the children, the demographic variables of the child, as well as the characteristics of their primary caregivers and parents (Table 3). Children who had been hospitalised were about 12 times more likely to die than those who were not; admittance to hospital was significantly associated with death, as was initiation of the antiretroviral treatment with a severely compromised immune system, that is, when the CD4 count was less than 15%. Although not significant, to be male and younger than 2 years old, was associated with clinical and immunological improvement. Nonetheless, children younger than 2 years were more likely to die than older children but the difference was not statistically significant. Similarly, when the primary caregiver was the grandmother, children were 3.3 times more likely to die than when cared for by other people but, again, the difference was not statistically significant. With regard to survival, initiation of the treatment when the CD4 count was less than 15% was the most significant predictor of death (Table 4). Children who had been admitted to hospital demonstrated no clinical improvement, but did show immunological improvement that was not statistically significant; similarly, children with a low CD4 count showed clinical improvement but not immunological improvement. Moreover, when compared to male children, female children showed no clinical improvement or immunological improvement. The majority of the children who died were female; 10 out of 14 deaths were female.

**TABLE 2:** Clinical and immunological parameters from baseline to the sixth month of treatment.

Paired variables and parameters	Mean values		Paired Mean Difference			
		
	At baseline	At 6 months	*n*	Mean	s.d.	Sig
Weight at baseline and Weight at 6th month (kg)	12.9	14.7	168	1.8	1.2	0.000*
CD4 count at baseline and CD4 count at 6th month (%)	10.8	23.5	96	12.6	10.4	0.000*

*Source*: Author's original data

*n*, number of children; s.d., Standard Deviation.

* *p* < 0.05.

**TABLE 3:** Factors associated with clinical and immunological improvement amongst children in the sample.

Variables and outcomes	Clinical improvement		Immunological improvement	
		
	Odd Ratio	CI	Odd Ratio	CI
**Child**				
Hospital admission status (admitted versus not)	0.99	0.44, 2.22	1.85	0.49, 7.05
Baseline immune suppression status (< 15% versus > 15%)	2.15	1.01, 4.59*	0.13	0.03, 0.57*
Sex (female versus male)	0.88	0.46, 1.69	0.65	0.27, 1.53
Age category (< 2 years versus > 2 years)	2.73	0.88, 8.47	3.36	0.40, 28.11
**HIV status**				
Mother (positive versus unknown)	0.89	0.44, 1.77	1.54	0.63, 3.78
Both parents (both positive versus discordant)	0.34	0.12, 1.02	1.11	0.27, 4.51
Both parents (both positive versus both unknown)	0.12	0.05, 0.28	0.56	0.16, 1.89
Father (positive versus unknown)	0.47	0.23, 0.98*	0.78	0.30, 1.98
**Living status**				
Father (dead versus alive)	0.93	0.47, 1.81	1.12	0.45, 2.80
Both parents (both dead versus one alive)	0.78	0.29, 2.04	1.54	0.36, 6.46
Both parents (both dead versus both alive)	0.76	0.28, 2.02	0.85	0.19, 3.78
Mother (dead versus alive)	0.89	0.46, 1.74	0.19	0.71, 4.93
**Primary Caregiver**				
Grandmother versus others	1.17	0.49, 2.77	1.11	0.36, 3.40
Mother versus grandmother	0.92	0.37, 2.26	0.84	0.26, 2.70
Mother versus others	1.09	0.58, 2.08	0.82	0.35, 1.93

*Source*: Author's original data

CI, Confidence Interval.

* *p* < 0.05.

**TABLE 4:** Factors associated with death amongst children in the sample.

Variables and outcomes	Death	
	
	Odd Ratio	CI
**Child**		
Hospital admission status (admitted versus not)	11.55	3.39–39.41*
Baseline immune suppression status (< 15% versus > 15%)	**	**
Sex (female versus male)	1.98	0.59–6.58
Age category (< 2 years versus > 2 years)	2.9	0.83–10.12
**HIV status**		
Mother (positive versus unknown)	1.12	0.34–3.73
Both parents (both positive versus discordant)	1.08	0.22–5.35
Both parents (both positive and both unknown)	1.09	0.17–7.05
Father (positive versus unknown)	0.84	0.22–3.18
**Living status**		
Father (dead versus alive)	0.15	0.02–1.17
Both parents (both dead versus one alive)	1.08	0.11–10.87
Both parents (both dead versus both alive)	0.23	0.05–1.12
Mother (dead versus alive)	0.72	0.22–2.39
**Primary caregiver**		
Grandmother versus others	3.27	0.93–11.50
Mother versus grandmother	0.37	0.09–1.40
Mother versus others	0.94	0.32–2.80

*Source*: Author's original data

CI, Confidence Interval.

* *p* < 0.05

** OR could not be calculated, there was no (zero) deaths in children with CD4 count > 15%.

With regard to the influence of caregivers and parents on the outcomes of treatment, the only factor that was significantly associated with clinical improvement was the father's unknown HIV status. A similar status for the mother was associated with immunological improvement but the difference was not statistically significant (Table 3). Caregiving by the grandmother was associated with some improvement in the clinical and immunological status but the difference was also not statistically significant. When compared to other caregivers, however, the children cared for by the grandmothers were threefold more likely to die, but the difference was again not statistically significant (Table 4). Similarly, when both parents were alive and when the father was alive, the children were more likely to survive, although the difference was not statistically significant.

At the end of the first year on antiretroviral treatment, 14 children had died. Of those who died, the majority were cared for by their mothers, whose HIV-positive status was known, and whose fathers were alive although their HIV status was not known (Table 5). The causes of the 14 deaths were recorded as well as infectious diseases, adverse events such as severe anaemia, and severe dehydration, severe malnutrition, and in a few cases the cause of death was not established.

**TABLE 5:** Characteristics of caregivers and parents of dead children.

Variables	*f*	%
**Primary caregiver (*n* = 14)**		
Mother	7	50.0
Aunt	1	7.1
Grandmother	4	28.6
Father	0	0.0
Others (Both parents, sister, grandfather, guardian)	2	14.3
**Living status Mother (*n* = 13)**		
Alive	9	69.2
Dead	4	28.6
**Living status Father (*n* = 12)**		
Alive	11	91.7
Dead	1	8.3
**HIV status Father (*n* = 14)**		
Father's status unknown	11	78.6
Father HIV-positive	3	21.4
**HIV status Mother's HIV status (*n* = 14)**		
Mother's status unknown	4	28.6
Mother HIV-positive	10	71.4

*Source*: Author's original data

*n*, given as a means of number; *f*, frequency.

## Discussion

Many factors influence the outcomes of antiretroviral treatment in children. One of the most important factors is that children younger than 6 years depend on their caregivers for all their needs, including accessing and adhering to treatment.^5, 6^ Whilst it is well-established that the socio-economic status of parents influences the health status of the parents as well as of their children;^7, 8^ this study investigated whether various categories of caregivers, the HIV status and the living status of parents, also influenced the outcomes of treatment and in particular mortality.

The majority of children was cared for by their mothers, and by close relatives such as aunts and grandmothers. The nature of the relationship between the child and the caregiver, therefore, suggests that people who were less likely to neglect the children cared for them. Indeed, based on the analysis, this closeness did not result in improved outcomes as would have been expected. This finding is contrary to reports from a study in Malawi where children cared for by their own parents fared much better than others.^9^ When the characteristics of parents are considered, it is encouraging that the HIV status of the majority of parents was known. The knowledge of their sero-status, however, had an ambivalent effect because the majority of children that died had mothers who were known HIV-positive but whose fathers’ HIV status was unknown. This finding suggests that the knowledge of the HIV status of the mother did not translate into a protective effect on the children. Perhaps the mothers were too sick to care for their children. Moreover, the majority of children that died had at least one parent still living. This finding concurs with a report by Gregson and colleagues^10^ who indicated that maternal orphans are likely to suffer more adverse health effects such as high proportions of HIV infections, other sexually transmitted infections, and pregnancies amongst teenage girls in eastern Zimbabwe. In this study, when both parents were alive or when the father was alive, the children were more likely to survive. This finding suggests that the presence of both parents had some positive influence.^11, 12^ It also concurs with reports from other studies that indicated that paternal death affects the economic environment of the household, maternal mood, as well as a number of other health and psychological outcomes.^13^ In addition, it has been established that fathers’ involvement in feeding decisions has been associated with increased positive outcomes such as the uptake of exclusive breastfeeding.^14^

With regard to the characteristics of the children, it has been found that their demographic profile has little influence on the outcomes, but their clinical status, in particular their immune status, was the most influential factor in their survival. In this study, children who started treatment when their immune status as measured by the CD4 count was lower than 15%, were most likely to show a significant clinical and immunological improvement but also were most likely to die. This finding concurs with reports from other investigators.^10, 11^ Remarkably, there were no deaths amongst children whose CD4 count was more than 15%. This finding supports the view that the antiretroviral treatment should be initiated before the immune system is too compromised.^12, 13, 14^ Hence, in line with the findings in this study, it is plausible to state that children whose parents were alive, were less likely to die because they may have been brought to access treatment earlier rather than later. This finding is consistent with the view that the father's involvement has the potential to impact positively on child development, child survival and health.^15^

The above findings suggest that the characteristics of caregivers and parents may influence the outcomes of antiretroviral treatment when interacting with the children's own clinical and immunological status. Given the design of this cross-sectional study, however, causal relationships could not be established. There is a need for more prospective studies with large sample sizes to investigate parents’ relationships and characteristics that influence treatment outcomes.

## Conclusion

The nature of relationships between caregivers and children on antiretroviral treatment, and the HIV status as well as the living status of their parents, seem to have little positive influence on the clinical, immunological, and survival outcomes of the treatment. More studies are needed to investigate other characteristics and relationships that may influence the outcomes of treatment.
